# Telling the truth to seriously ill children: Considering children's interests when parents veto telling the truth

**DOI:** 10.1111/bioe.13048

**Published:** 2022-05-19

**Authors:** Lynn Gillam, Merle Spriggs, Maria McCarthy, Clare Delany

**Affiliations:** ^1^ Children's Bioethics Centre Royal Children's Hospital and University of Melbourne Parkville Victoria Australia; ^2^ Murdoch Childrens Research Institute Parkville Victoria Australia; ^3^ Royal Children's Hospital Parkville Victoria Australia

**Keywords:** child, communication, ethics, parents, patient, truth disclosure

## Abstract

How should clinicians respond when parents will not allow their child to know the truth about their medical condition and treatment? There is wide consensus amongst clinicians and ethicists that children should be given “honest” information delivered in a developmentally appropriate manner. However, the basis in ethical theory is not clear, especially for pre‐adolescents. These children are old enough to understand some information, but are not yet “mature minors” capable of making their own health care decisions. We take the position that thinking in terms of a child's “right to know” is not the most helpful in dealing with the ethical complexity of these situations. We propose that questions of truth‐telling are best addressed in terms of how a child's interests are promoted or set back by being told the truth. Our first step is to give an account of the concept of children's interests in general. Then we relate that account specifically to truth‐telling. In doing so, we use a relatively straightforward hypothetical but realistic case, in order to illustrate how ethical deliberation using interests would proceed. The case is not intended to be particularly contentious or difficult, so that the focus is on the nature of the ethical reasoning, rather than any complexities of the case.

## 1 INTRODUCTION

*Laura is a 7‐year‐old girl with bone cancer in her leg. Curative treatment for her cancer requires amputation of her leg. Laura is relatively well, and has been discharged from hospital, with surgery scheduled in 2 weeks’ time. Laura has coped well with the investigations and treatment she has had so far, and she has strong relationships with her parents and family. Laura's parents are relieved that she is so settled, and decide that they do not want her to be told about amputation until the day of the procedure. This is contrary to the usual practice of the clinical team, who are taken aback by the parents' firm instruction to them not to speak with Laura about the amputation*.

“Truth‐telling” or disclosure of information to children and adolescents with serious childhood illnesses is an issue that has gained increasing attention over recent decades. The current widely held position is that children should be presented with “honest” information delivered in a “developmentally appropriate” manner.^1^ Whilst we accept this general position, we propose that its ethical foundations need greater scrutiny. Over the past several decades, the consensus view about “truth‐telling” to children and adolescents with serious childhood illnesses has moved through significantly different phases.^2^ In the 1950s, recommendations favored a protective approach to shield children (and indeed adults) from the harmful effects of being given “bad news.”^3^ This view then shifted towards a more open approach to disclosure, and then further, in the 1980s towards an “always tell” approach.^4^ The latter shift reflects the influence of the United Nations Convention on the Rights of the Child,^5^ where truth‐telling and open discussion are seen as an important part of protecting a child's rights.^6^

The question of what constitutes “the truth” about a serious medical condition in a child is of course somewhat problematic, when treatment and prognostic information is probabilistic, uncertainty abounds, and there is the possibility of new treatment breakthroughs.^7^ Nevertheless, even if we cannot ever “fully grasp or express the whole picture,” it is possible to “speak truthfully.”^8^ This is the sense in which we use the term “truth‐telling.” We agree with Higgs that one can distinguish between the abstract concept of “truth,” which may be impossible to establish and “*telling* the truth” or speaking “truthfully”, where the intention is “to convey what we understand” and not to mislead.^9^ Speaking truthfully can include uncertainty, “likelihoods,” “informed guesses” (if they are acknowledged to be guesses) and saying “I do not know.”^10^ Truth‐telling is disclosing openly and honestly what one knows or understands, with whatever degree of uncertainty and interpretation is attached to that.

In this paper, we revisit the question of telling the truth to children through the lens of a case such as the one above, where parents request or demand that their child not be told the truth. We put forward the position that thinking in terms of a child's “right to know” is not the most helpful in dealing with the ethical complexity of these situations. We propose that questions of truth‐telling are best addressed in terms of how a child's interests are promoted or set back by being told the truth. The case is intended to be relatively straightforward, rather than particularly contentious or difficult. It has been chosen in order to allow us to focus on the nature of the ethical reasoning, rather than on what the ethically appropriate outcome should be. This case, and others like it, highlight the need to be able to give ethical reasons for telling the truth to a particular child in particular circumstances, and also to contemplate possible ethical reasons not to tell. When parents make such a request, they are surely owed at least proper consideration for their preferences. They should be asked their reasons; these reasons should be fully considered, and if the clinicians or clinical ethics team decides to act against their wishes, then parents should be given a full explanation that addresses their reasons. They are owed this even if in the end the ethical decision that the child should be told is relatively clear‐cut. As suggested by our case, our focus is on younger children: children who are old enough to understand some information, but are not yet “mature minors” capable of sufficient understanding to make their own health care decisions. These children may be capable of having input into decision‐making in various ways (such as expressing concerns, asking questions, saying what matters to them), but do not have the cognitive capacities and psychological maturity to make a fully autonomous decision of their own, which would constitute a valid informed consent to or refusal of medical treatment. This age group is of special interest, because a primary reason driving truth‐telling for competent adults (that they need the truth in order to make an informed decision), does not apply.

Whilst the wide consensus about giving children “honest” information in a developmentally appropriate manner applies to these younger children (primary‐school aged children, approximately 5–11 years), there has been little detailed ethical analysis to support this. A recent paper from our group^11^ surveyed the ethical and psychosocial literature to identify reasons given in favor of telling the truth to “immature minors.” This paper identified six major claims about why younger children should be told the truth. Broadly speaking, these claims fall into three categories. The first two are tangible claims: firstly, that truth‐telling will promote the child's well‐being at the time of their illness or hospitalization; secondly, that it will lay a foundation that will help them in the future when they are older and need to be able to make their own decisions. The third category relates to intangible matters—that truth‐telling respects the child as a person, or instantiates the ethically proper relationship between clinician and child. Interestingly, claims about the younger child's intrinsic “right to know” were not prominent.

In relation to younger children, there are limited empirical studies to support truth‐telling as beneficial. Much of the research on children's desire for information, and the beneficial effects of being given information, has been conducted with adolescent patients, whose increasing autonomy and cognitive ability make the ethical basis for truth‐telling relatively more straightforward. Less is known about younger, pre‐adolescent children. Coyne et al.'s studies on information‐sharing and shared decision‐making (which included children as young as 7 years), though not directly about truth‐telling, showed that children with cancer wanted to receive information about their treatment,^12^ and trusted their parents to provide but also to “buffer” that information.^13^ Coyne et al.'s findings paint a somewhat different picture from Levetown and the Committee on Bioethics’ earlier summation that studies to that point (2008) indicated pre‐adolescent children were “passive recipients” of medical care, with almost no involvement in discussions about their care planning.^14^ This may suggest that there has been some degree of change quite recently, or it may simply be that the settings are different. Overall, the number of studies so far is small, meaning that it is not possible to make conclusive statements about the effects on younger children of telling them the truth.

Ultimately, then, the empirical evidence does not offer any compelling reasons for or against telling the truth to younger children. Further, the basis in ethical theory for the standard reasons for truth‐telling to younger children has not been explicated in any detail. Currently, a lot of the normative work is being done by very general ideas that truth‐telling is beneficial and respectful, and that the child has a right to know the truth. Applying these ideas to specific cases where parents veto truth‐telling leaves a great deal open to endlessly contestable interpretation, or simply to claim and counter‐claim. In particular, we suggest that thinking in terms of a child's “right to know” is not particularly helpful in these situations. In this paper, we propose another approach, which focuses on the interests of the child. The question about whether a child should be told the truth, we suggest, should be addressed by asking whether telling the truth would promote or set back the child's interests. This approach requires a nuanced understanding of what constitutes a child's interests, both in general and in particular situations, such as diagnosis of a life‐threatening condition such as cancer, approaching major medical procedures with life‐changing outcomes, and nearing the end‐of‐life. It also involves careful consideration of how being told the truth relates to these interests, for each particular child in their unique set of circumstances.

## 2 RIGHTS AND INTERESTS—RATIONALE FOR OUR METHODOLOGY

Talking in terms of rights has been a common way to discuss what should be done in cases involving disclosure of information to children with serious medical conditions; for example, a child's right to information or a child's right to know. The concept of children's rights is philosophically complex. Firstly, there are complexities in a rights‐based approach to ethics, just as there are in any approach that recognizes plural and potentially conflicting values. As Archard explains, all rights are not equally important and do not all entail the same level of obligation on others: an individual can have rights that are in tension with each other. Moreover, the rights of one individual do not automatically trump the rights of others. In short, any particular right of one person does not override all other considerations.^15^ Secondly, the concept of the rights of *children* adds another layer of complexity. Children are often not able to voice their rights, which is precisely the reason that children's rights theorists believe acknowledgment of their rights (such as a right to be heard) is so important.^16^ However, in the same vein, children often cannot articulate whether or not they wish to claim their rights. Rights are entitlements to have something, *if one wants it*.^17^ A right to know is a right to know something *if one wants to know*. For some rights, such as the right to life, it is very reasonable to assume that a person wants to claim this right; but in regard to the right to know in health care, it is widely acknowledged that some people may not wish to know some information.^18^ For instance, some people may not wish to know genetic or prognostic information.^19^ Upholding a child's right to know also means upholding their right not to know: the critical factor in determining which right to uphold is whether the child wants to know. Unfortunately, this may often be very difficult to ascertain, more so than it is for adults, at least without explicitly asking the child if they have any questions. Further, offering the truth, as Freedman argues for in adults,^20^ may well not be as effective for children, due to their common reluctance to speak up, and their tendency to defer to their parents. In cases such as Laura's, parents do not want their child to be offered any information: that is part of what they are asking from clinicians.

Given all this, we suggest that a focus on interests is a more useful way of thinking about contested cases of telling the truth to children such as Laura. The concept of “interests” is still complex in some of the ways in which rights are complex, since a person has multiple interests, all of which might not equally be able to be fulfilled in particular circumstances; and the interests of different individuals can be in tension.^21^ However, thinking in terms of interests is somewhat more straightforward, in that it goes directly to what really matters for the child, rather than first having to consider conceptual or theoretical questions such as what kind of right is at stake (positive or negative) or what correlative duty it entails, and for whom. The question of the child's interests focuses attention immediately on the child, and how knowing or not knowing the truth will impact on that particular child. Thinking about interests also avoids the pitfall of deploying the “right to know” simplistically, as an absolute right that trumps all other considerations.^22^ Interests are not typically conceived of as absolutes, or trumps, so do not suffer from this rhetorical drawback. For these reasons, we propose that questions about truth‐telling to children are most effectively framed in terms of whether and how truth‐telling would promote or undermine a child's interests.

## 3 THE CONCEPT OF CHILDREN'S INTERESTS

The concept of “the child's best interests” lies at the core of pediatric clinical ethics.^23^ However, it is also widely acknowledged as a contestable concept, or at least a contestable standard for decision‐making about a particular child in their particular circumstances.^24^ There can be competing views about what counts as being in a child's best interests, primarily because children have a number of interests (as do adults), which can be affected in different ways and to different degrees by any one course of action.^25^ We are proposing that the appropriate ethical question to ask is whether telling the truth is in the child's best interests, whilst at the same time emphasizing that the answer to this is not at all straightforward. Deeper exploration of the concept of children's interests is required. On a standard account, the term “a person's interests” refers to a person's well‐being or “what makes a person's life go well.”^26^ This general definition holds for all persons, adults or children.

Children's interests, however, are conceptually different to the interests of an adult. The dominant or popular view in contemporary ethics about what makes an adult's life go well ties it to the satisfaction of their desires or preferences.^27^ An adult's interests amount to the things that each person desires, or more precisely, as DeGrazia's widely accepted definition states, would desire if rational and well‐informed.^28^ These desires and preferences are revealed in people's choices, making it possible to measure, weigh or rank the value of preference satisfaction.^29^ Objective list theories are the other contenders for what makes a life go well. Well‐being, on an objective‐list account, consists in things that are good for anyone, even though a particular person may not realize it, desire it, or find it satisfying or enjoyable.^30^ Although the desire‐based account of well‐being is the dominant view,^31^ there is a sense in which the contender accounts of well‐being are not clear‐cut. Some theorists have suggested that the current division of theories “may be inadequate.”^32^ Both Crisp and Skelton propose that a hybrid account may work,^33^ while DeGrazia suggests that a “plausible theory” could be an objective list theory that makes concessions to subjective theories.^34^

For a child however, a desire‐based account of interests is arguably not appropriate. By their very nature, children have a very limited capacity to take a holistic view of their own lives or to see their lives extending into the future and considering what will make their life go well over the longer term. Whilst looking ahead in this way can be difficult enough for adults, there is an added confounding factor for children: in attempting to look ahead, they must imagine themselves as an adult, a qualitatively different being from the child they are now.^35^ Further, their values (their sense of what ultimately matters to them) are not formed yet.^36^ For these reasons, it is not justifiable to equate their interests with their current desires. As Skelton argues, children are unlikely to have a “set of desires” that will “capture all of what matters” to their life going well.^37^ Binik explains it this way:

Children do not have fully formed wills. It is for this reason that we consider it appropriate for parents and guardians to make decisions on behalf of children and do not trust children to make overriding determinations about what is in their own interests. It follows that it should seem more palatable to say that certain things are good or bad for a child irrespective of whether she desires these things (although we should not necessarily disregard a child's likes and dislikes).^38^

For children, then, a more reasonable position is to adopt an objective‐list account of interests. This sort of account is not controversial in the way that an objective list of adult's interests would be controversial.^39^ Adopting the objective‐list account does not mean that children's wishes are ethically irrelevant, or that only their long‐term interests matter. As we will show below when discussing Laura's case further, short‐term interests can be highly relevant in relation to truth‐telling.

### 3.1 An objective‐list account of child‐specific interests

An objective‐list account of children's interests would need to encapsulate two dimensions: things that promote a child's well‐being while they are in the stage of childhood, and things that will provide the foundation for their life going well in their own terms as an adult. This meets Campbell's condition on accounts of children's well‐being that they must have an “intuitive fit” with a plausible account of adult well‐being, and with the slow transition from childhood to adulthood.^40^ While the literature on what constitutes a child's well‐being as opposed to an adult's well‐being is described as being “in its infancy,”^41^ there have been some attempts to articulate the components of a child‐specific list of interests that identify broad areas of consensus amongst different accounts of human goods.^42^ Some of these accounts, while offering valuable insights about children's well‐being, have been developed for quite specific contexts, such as the involvement of children in research. As noted by Campbell in his commentary on Binik's paper, it is preferable to develop an account of children's interests that is not so specifically targeted to the needs of a particular context.^43^ On the other hand, objective‐list accounts of well‐being do depend, to some extent, on the particular context to guide which interests to emphasize or prioritize. Our aim was to use as a starting point a list of children's interests that broadly has the health care context in mind, but is not specifically directed toward the issue of truth‐telling (so that our argument would not be question‐begging). While there is no single list that is universally agreed on and in use, we regard the list developed by Janet Malek as the best current candidate.^44^

Malek's list was designed to be practically applicable: its purpose is to help clinicians “make judgments” about treatments “that best promote children's well‐being.”^45^ However, it is not simply pragmatically targeted to that purpose. It provides a quite general descriptive account, based on the “combined conclusions” or “collective wisdom” of three credible sources from the political, medical and philosophical literature:^46^ (a) The United Nations’ Convention on the Rights of the Child;^47^ (b) *The irreducible needs of children*, by Brazelton (a pediatrician) and Greenspan (a child psychiatrist)^48^; and, (c) Martha C. Nussbaum's list of human capabilities that are of central importance in any human life.^49^ None of these accounts, by themselves, provide an adequate account of children's interests, but Malek has qualitatively analyzed the three accounts to produce a “defensible” description of children's interests.^50^

### 3.2 The interests of children: Malek's list

Malek's list of children's interests (see Table 1)^51^ presents a broad view of children's well‐being. The list includes items such as life, health and health care, basic needs, protection from neglect and abuse—things that a child needs to stay alive and healthy. Other items include play and pleasure, interaction, emotional development, cognitive development, expression and communication, identity, sense of self and autonomy. These are factors that will both make a child's life go well as a child, and also provide the building blocks for life to go well in adulthood—the growth of a sense of self, identity and autonomy is a basis for having the sort of desires (rational, well‐informed, arising from one's own values) in adulthood that are envisaged by the widely accepted and most plausible desire‐based accounts of adult interests.^52^

**Table 1:** Malek's list: Interests of children.

1. Life. To live and to anticipate a life of normal human length.
2. Health and health care: To have good health and protection from pain, injury, and illness. To have access to medical care.
3. Basic needs: To have an adequate standard of living, especially to be adequately nourished and sheltered.
4. Protection from neglect and abuse: To be protected from physical or mental abuse, neglect, exploitation, and exposure to dangerous environments. To be secure that they will be safe and cared for.
5. Emotional development: To experience emotion and have appropriate emotional development.
6. Play and pleasure: To play, rest, and enjoy recreational activities. To have pleasurable experiences.
7. Education and cognitive development: To have an education that includes information from diverse sources. To have the ability to learn, think, imagine, and reason.
8. Expression and communication: To have the ability to express themselves and to communicate thoughts and feelings.
9. Interaction: To interact with and care for others and the world around them. To have secure, empathetic, intimate, and consistent relationships with others.
10. Parental relationship: To know and interact with their parents.
11. Identity: To have an identity and connection to their culture. To be protected from discrimination.
12. Sense of self: To have a sense of self, self‐worth, and self‐respect.
13. Autonomy: To have the ability to influence the course of their lives. To act intentionally and with self‐discipline. To reflect on the direction and meaning of their lives.

Interests come as an interrelated package, rather than a set of independent items. According to Malek, “a lack of any one of the interests can severely compromise a child's overall well‐being” and the “excessive promotion” of any one interest is not likely to advance overall well‐being.^53^ This fits in with the way that the interests on Malek's list do not have sharp definitional boundaries, but rather overlap in various ways. For instance, a child's interest in communicating fears and concerns (expression and communication) relies on information and interaction with parents and clinicians. A child's interest in the parental relationship involves interacting to having a secure, intimate and consistent relationship.

### 3.3 A cluster of children's interests: A new representation of Malek's list

**Figure 1:**
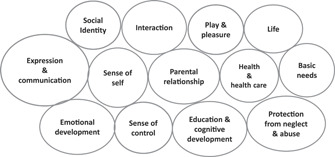
Cluster of children's interests.

In order to emphasize the interrelated nature of the various interests of children, we have chosen to represent Malek's list visually as a cluster of interests (see Figure 1). This also conveys more clearly that there is not a set hierarchical order of importance (the size of the shapes in the cluster diagram is not intended to convey anything about relative importance). Of course, life and basic needs could be regarded as primary or foundational, in the sense that a child needs to be alive and well enough to have experiences before any of the other interests come into play. However, it is worth noting that in many situations in which this account of children's interests might be used, life and basic needs are likely not to be of critical concern, because in the context, these can be assumed. It will be the other interests where most debate will focus.

According to Malek, her list is “open to revision.”^54^ We have slightly modified the names of two of the interests in order to ensure they are clearly relevant to younger as well as older children, and in the broad health care context. We have re‐named “autonomy” as “sense of control.” We agree with Malek's position that it is a good thing that children have the opportunity “to influence the course of their lives” and “reflect on the direction and meaning of their lives”^55^—that is, to the extent that they are capable of this and want to do it. However, we do not regard the term “autonomy” as the best one to represent these ideas. In an ethics context, where there are competing accounts of autonomy and where younger children would be regarded as non‐autonomous on accounts that focus on capacity to make decisions,^56^ this term risks an overly narrow interpretation. For this reason, we have opted for “sense of control,” which is intended to speak to a child's sense of agency in general, and not simply competence or capacity to make decisions about their own health care. We have also changed “identity” to “social identity,” which seems more appropriate in this context. Malek's explanation of “identity”^57^ as “having a connection to their culture” and “not be discriminated against” (presumably on the grounds of that cultural connection) again seems to suggest too narrow an interpretation. Many things other than culture are relevant to a child's developing sense of personal identity and understanding of where they fit into the world, so we have used the term “social identity” in an attempt to indicate this.

## 4 HOW TRUTH‐TELLING FITS IN WITH CHILDREN'S INTERESTS

Using the concept of children's interests provides a framework or language to think about truth‐telling, without making any prima facie assumption that truth‐telling is inherently a good thing for children. It is notable that Malek's list does not include any reference to truth‐telling. Our approach is to use the list of interests in the way Malek recommends, to facilitate identification and analysis of how an issue or situation bears on a child's interests, and provide a way to take a critical/reflective step back from initial judgments. This approach also, and very importantly, allows consideration of the individual child and their context.

Our suggestion, then, is that in the pediatric clinical setting, questions about truth‐telling can be framed in terms of whether and how it would promote or undermine children's interests. To demonstrate how this would work, we now return to the case we described at the beginning of this paper.

## 5 INTERESTS‐BASED ANALYSIS OF LAURA'S CASE

To briefly recapitulate, Laura has a serious medical condition, although she is not dying. Treatment for her bone cancer requires amputation but her parents do not want her to be told about the amputation until the day of the operation, which is 2 weeks away. Ethical deliberation about the delay in telling Laura the truth using the interests‐based approach involves two aspects: (a) identify which of the interests are most relevant, in what way; and (b) assess what impact temporary withholding of the truth would have on these interests. Some interests need not be factored in because they are not in question in this case. These include basic needs, health and health care, and protection from neglect and abuse—these are not at stake for Laura.

Laura's relationship with her parents is an important interest here. Not telling her about the amputation until the day of the operation will not necessarily have a negative impact on the relationship in the short‐term (in fact, her parents may believe that not telling will protect the relationship from damage), but it is likely to undermine her trust in her parents long‐term, once she finds out that they have known for 2 weeks and not told her. Her related interest in communication and expression will also be compromised. Not being able to communicate her thoughts and fears with her parents until the last few hours before the procedure means that she has missed out on the help her parents could have provided in allaying her fears and anxieties over a longer period of time.^58^

Similarly, not telling Laura about the amputation until the last moment will very probably undermine her interest in having “secure” and “consistent relationships” with the clinicians who are treating and caring for her.^59^ The clinicians will have no way to gain her trust or co‐operation in advance of the procedure.^60^ They will need to actively hide important information from her when they talk with her. So Laura will not be able to be fully involved in a genuine therapeutic relationship.^61^ This in itself undermines genuine interaction and relationship‐building; having to tell her at a late stage what they have known all along is likely to create an open rift.

Sense of self is another relevant interest for Laura. Losing a leg will surely affect her sense of self. Presumably, she would benefit from having time to process information about what is going to happen and why, so that she can begin to adjust to her new reality.^62^ Whilst this could be done after the procedure, having a head‐start would better promote her interest in having a secure sense of self. Sense of control is likely to be affected in a similar way. Without information ahead of the amputation, Laura's opportunity to be pre‐warned and prepare or adjust is severely limited. Her role will be essentially passive: things will happen to her, seemingly out of the blue; she will have no sense of control. Research shows that children who are informed about their condition and what is happening to them gain a sense of control, and a reduction in anxiety.^63^ They are also better able to understand their illness and have the things that matter to them taken into account.^64^ This point relates also to the interest in emotional development. Laura needs time to process information about her condition and to understand the need for amputation in order to grieve and adapt to the loss of her leg. She has an interest in developing her capacity to cope. When her parents delay telling her about the amputation until the day of the operation, she has little time to process the information and she misses the opportunity to learn from observing how others have coped. She also misses out on the possibility of finding acceptance and comfort from shared experiences with others.^65^

### 5.1 Summing up for this case

Our analysis suggests that overall, Laura's interests are likely to be undermined if she is not told about the amputation earlier, even though at first glance, delay in telling her might seem a much kinder option. Note that this conclusion depends on certain assumptions about Laura, her parents and the nature of their relationship. In particular, we have assumed that her parents would be willing and able to support her to work through fears and worries about amputation in the time before surgery, so that by the time she comes to hospital she has developed some equanimity, coping strategies and a positive way of seeing the future. We acknowledge, however, that this would not necessarily be so for all children. For a child who is highly anxious, whose fears increase rather than decrease with time to think and talk, telling earlier may actually set back their interests. We suspect that this would be an uncommon situation, but that is an empirical question. The key thing is to consider the effects on the interests of each child individually.

## 6 CONCLUSION

We have proposed that the ethical question of whether a child should be told the truth about their medical condition or treatment is best answered by considering how truth‐telling would affect that child's interests, either positively or negatively. While current accounts of children's interests do not include “being told the truth” as a primary interest of children, we have argued that truth‐telling relates to many of the interests of children that are included in these accounts. In the health care setting, this is an important reminder that clinicians aiming to make decisions that are in the best interests of a child need to consider not just “medical interests,” narrowly conceived, but rather how their actions would affect all of the child's interests (their “overall interests”).^66^ Our argument in this paper could be taken as drawing attention to the “informational interests”^67^ of children, alongside their “medical” interests. A better way to express this, we suggest, is that we are drawing attention to the fact that what clinicians *say* to children can affect their interests just as much as the medical procedure they perform on children. The ethical question of what information to provide to the child should, in general terms, be approached in the same way as the ethical question of what medical treatment to provide: by considering what impact each of the various options will have on the child. This type of reflection provides a more structured and practical way for a clinician to consider whether, when and how much information to give to a child than merely telling the clinician to ensure they respect a child's right to the truth.

We have re‐framed the standard list of interests as a non‐linear “cluster” of interests, emphasizing that separately named items on a list of interests are connected and overlapping, in that they can be affected in related ways by a particular action, whether it be truth‐telling, medical treatment, or something else. The aim is not to argue for one interest taking precedence over others (as might be implied by presenting a list of interests), since lists are inherently ordered in some way, but to develop a multifactored overall evaluation. When interests are understood in this way, it is easy to see how their relative importance and emphasis may change depending on the particular context and situation. We have suggested that in many situations where children are seriously ill, considering the effects of withholding information on the full cluster of their interests will lead to the conclusion that their interests will be best promoted by truth‐telling. In particular, in the case of Laura discussed here, we have proposed that when the truth is withheld, a child is deprived of the opportunities to grieve and to process, to seek comfort from the supportive relationships that they have, and to develop some sense of control over what is happening. These lost opportunities are a set‐back to the child's interests, even if the child is not aware of the lost opportunity, and does not feel any regret or distress at it.

However, there is not one overall answer to the question of whether a child should be told the truth about their medical condition and treatment. The conclusion we came to about telling the truth to Laura applied to the child we presented her to be, in that particular situation. Whilst it might seem an easy and obvious conclusion that all children should be told the truth about their upcoming medical procedure in a timely fashion, this should not simply be assumed. The particularities of the child, their personal characteristics and the social context matter.

Moreover, if Laura's medical situation were different, the interests‐based approach to truth‐telling would need to take this into account, too. Imagine that despite the amputation, Laura's cancer was not cured as expected, and 2 years later she had untreatable metastatic bone cancer. We might not be surprised to hear that her parents do not want her to be told that she is going to die within weeks. In this circumstance, which many would regard as more ethically challenging and contentious, taking the interests‐based approach to truth‐telling would mean asking the same questions about how disclosure of this information would affect all of Laura's interests, in comparison to not disclosing it. The answers might turn out differently from telling her about the planned amputation, or they might not. It would depend on what is known from research about the experiences and wishes of dying children in general, but also on what we know about Laura and her family in particular. These complex questions cannot be addressed within the scope of this paper.

In the meantime, the key message of this paper is that the cluster of interests approach fosters careful and nuanced deliberation about which interests are affected, and to what degree, by an action. This provides guidance for clinicians responsible for ascertaining whether telling the truth would promote a particular child's interests in a particular situation.

## CONFLICTS OF INTEREST

The authors declare no conflicts of interest.

## Footnotes

1 Masera, G., Chesler, M. A., Jankovic, M., Ablin, A. R., Ben Arush, M. W., Breatnach, F., McDowell, H. P., Eden, T., Epelman, C., Fossati Bellani, F., Green, D. M., Kosmidis, H. V., Nesbit, M. E., Wandzura, C., Wilbur, J. R., & Spinetta, J. J. (1997). SIOP working committee on psychosocial issues in pediatric oncology: Guidelines for communication of the diagnosis. *Medical and Pediatric Oncology, 28*(5), 382–385; British Medical Association. (2001). *Consent, rights and choices in health care for children and young people*. BMJ Books; Harrison, C. (2011). Fidelity and truthfulness in the pediatric setting: Withholding information from children and adolescents. In D. Diekema, M. R. Mercurio, & M. B. Adam (Eds.), *Clinical ethics in pediatrics: A case‐based textbook* (pp. 32–36). Cambridge University Press; Mack, J. W., & Joffe, S. (2014). Communicating about prognosis: Ethical responsibilities of pediatricians and parents. *Pediatrics, 133*(Suppl 1), S24–S30; American Academy of Pediatrics. (2016). Informed consent in decision‐making in pediatric practice. *Pediatrics, 138*(2), 1–7.

2 Sisk, B. A., Bluebond‐Langner, M., Wiener, L., Mack, J., & Wolfe, J. (2016). Prognostic disclosures to children: A historical perspective. *Pediatrics, 138*(3), 1–19.

3 Ibid: 1.

4 Ibid: 5.

5 United Nations. (1989). *Convention on the rights of the child*. https://www.ohchr.org/Documents/ProfessionalInterest/crc.pdf

6 Harrison, op. cit. note 1.

7 Coyne, I., Amory, A., Gibson, F., & Kiernan, G. (2016). Information‐sharing between healthcare professionals, parents and children with cancer: More than a matter of information exchange. *European Journal of Cancer Care, 25*(1), 141–156.

8 Higgs, R. (2016). On telling patients the truth. In H. Kuhse, U. Schuklenk, & P. Singer (Eds.), *Bioethics: An anthology* (pp. 621–627). John Wiley & Sons Inc., p. 623.

9 Ibid: 623.

10 Ibid: 623.

11 Hudson, N., Spriggs, M., & Gillam, L. (2019). Telling the truth to young children: Ethical reasons for information disclosure in paediatrics. *Journal of Paediatrics and Child Health, 55*(1), 13–17.

12 Coyne, I., Amory, A., Kiernan, G., & Gibson, F. (2014). Children's participation in shared decision‐making: Children, adolescents, parents and healthcare professionals' perspectives and experiences. *European Journal of Oncology Nursing, 18*(3), 273–280.

13 Coyne et al., op. cit. note 7.

14 Levetown, M., & the Committee on Bioethics. (2008). Communicating with children and families: From everyday interactions to skill in conveying distressing information. *Pediatrics, 121*(5), e1441–e1460, p. e1446.

15 Archard, D. (2014). *Children: Rights and childhood*. Routledge.

16 Archard, D. (2018). Children's rights. In E. N. Zalta (Ed.), *The Stanford encyclopedia of philosophy*. Metaphysics Research Lab, Stanford University. Retrieved from https://plato.stanford.edu/archives/win2018/entries/rights-children/

17 Wenar, L. (2015). Rights. In E. N. Zalta (Ed.), *The Stanford encyclopedia of philosophy*. Metaphysics Research Lab, Stanford University. Retrieved from https://plato.stanford.edu/archives/fall2015/entries/rights/

18 In arguing for an interests‐based approach to truth‐telling for younger children, rather than a rights‐based one, we did not intend to endorse the idea that a rights‐based analysis would necessarily be more appropriate for older children and adults. Changing over to a rights‐based approach for older children would raise the obvious question about how to know when to switch—at what development stage, and based on what markers of stage? It could be argued that rights are based ultimately on interests, so in fact there would be no major discontinuity; just a gradual change in the understanding of the nature of children's interests as children develop and their interests begin to individuate to some degree, as they form a unique set of desires for their own lives. However, this is a complex matter, which unfortunately we cannot address in the necessary depth in this paper.

19 Chadwick, R., Levitt, M., & Shickle, D. (2014). *The right to know and the right not to know: Genetic privacy and responsibility*. Cambridge University Press.

20 Freedman, B. (1993). Offering truth: One ethical approach to the uninformed cancer patient. *Archives of Internal Medicine, 153*(5), 572–576.

21 Wenar, op. cit. note 17.

22 Van Hooft, S., Gillam, L., & Byrnes, M. (1995). *Facts and values: An introduction to critical thinking for nurses*. MacLennan & Petty, p. 222.

23 Kopelman, L. (1997). The best‐interests standard as threshold, ideal, and standard of reasonableness. *The Journal of Medicine and Philosophy, 22*, 271–289; Fleischman, A. (2016). *Pediatric ethics: Protecting the interests of children*. Oxford University Press.

24 Allan, S., & Blake, M. (2014). *The patient and the practitioner*. LexisNexis Butterworths; Fleischman, op. cit. note 23.

25 McDougall, R., Gillam, L., Kerruish, N., & Snelling, J. (2016). Within the ZPD: Focusing on harm and children's interests. In R. McDougall, C. Delany, & L. Gillam (Eds.), *When doctors and parents disagree: Ethics, paediatrics and the zone of parental discretion* (pp. 25–40). The Federation Press.

26 DeGrazia, D. (1995). Value theory and the best interests standard. *Bioethics*, *9*(1), 50–61; Malek, J. (2009). What really is in a child's best interest? Toward a more precise picture of the interests of children. *Journal of Clinical Ethics, 20*(2), 175–182; Wendler, D. (2010). *The ethics of pediatric research*. Oxford University Press; Binik, A. (2014). On the minimal risk threshold in research with children. *American Journal of Bioethics, 14*(9), 3–12; Skelton, A. (2015). Utilitarianism, welfare, children. In A. Bagattini & C. Macleod (Eds.), *Nature of children's well‐being* (pp. 85–103). Springer; Skelton, A. (2019). Children and well‐being. In A. Gheaus, G. Calder, & J. De Wispelaere (Eds.), *The Routledge handbook of the philosophy of childhood and children* (pp. 90–100). Routledge.

27 Crisp, R. (2017). Well‐being. In E. N. Zalta (Ed.), *The Stanford encyclopedia of philosophy*. Metaphysics Research Lab, Stanford University. Retrieved from https://plato.stanford.edu/archives/fall2017/entries/well-being/; Skelton (2015), op. cit. note 26.

28 DeGrazia, op. cit. note 26; the term “rational” here is used in a technical sense, showing how desires are defined in desire‐based accounts of interests. This is a different context from any discussion of “rationality” in relation to decisional competence. We are not implying that “rationality” is required for a person's health care decision to be regarded as autonomous or deserving of respect, either for children or adults.

29 Crisp, op. cit. note 27, p. 6.

30 DeGrazia, op. cit. note 26, p. 55; Kagan, S. (1992). The limits of well‐being. *Social Philosophy & Policy, 9*(2), 169–189, p. 170.

31 Crisp, op. cit. note 27, pp. 6–7; Skelton (2015), op. cit. note 26, p. 96.

32 DeGrazia, op. cit. note 26, p. 55.

33 Crisp, op. cit. note 27, p. 8; Skelton (2015), op. cit. note 26.

34 DeGrazia, op. cit. note 26, p. 55.

35 Skelton (2015), op. cit. note 26, p. 98; Campbell, S. M. (2014). Standards for an account of children's well‐being. *American Journal of Bioethics, 14*(9), 19–20, p. 19.

36 Skelton (2015), op. cit. note 26, p. 97; Mullin, A. (2007). Children, autonomy and care. *Journal of Social Philosophy, 38*(4), 536–553, p. 536.

37 Skelton (2015), op. cit. note 26, pp. 96–97.

38 Binik, op. cit. note 26, p. 8.

39 Ibid.

40 Campbell, op. cit. note 35, p. 19.

41 Skelton (2019), op. cit. note 26, p. 94.

42 Malek, op. cit. note 26; Wendler, op. cit. note 26; Binik, op. cit. note 26; Skelton (2015), op. cit. note 26; McDougall et al., op cit. note 25; Skelton (2019), op. cit. note 26.

43 Campbell, op. cit. note 35, p. 20.

44 Malek, op. cit. note 26.

45 Ibid: 175.

46 Ibid: 179.

47 United Nations, op. cit. note 5.

48 Brazelton, T. B., & Greenspan, S. I. (2000). *The irreducible needs of children: What every child must have to grow, learn, and flourish*. Perseus.

49 Nussbaum, M. C. (2000). *Women and human development: The capabilities approach*. Cambridge University Press.

50 Malek, op. cit. note 26.

51 Ibid: 180.

52 DeGrazia, op. cit. note 26.

53 Malek, op. cit. note 26, p. 180.

54 Ibid: 179.

55 Ibid: 180.

56 Buchanan, A. E., & Brock, D. W. (1990). *Deciding for others: The ethics of surrogate decision making*. Cambridge University Press, pp. 84, 229; Mullin, op. cit. note 36.

57 Malek, op. cit. note 26, p. 180.

58 Last, B. F., & van Veldhuizen, A. M. H. (1996). Information about diagnosis and prognosis related to anxiety and depression in children with cancer aged 8–16 years. *European Journal of Cancer, 32*(2), 290–294; Masera et al., op. cit. note 1; British Medical Association, op. cit. note 1; Badarau, D. O., Wangmo, T., Ruhe, K. M., Miron, I., Colita, A., Dragomir, M., Schildmann, J., & Elger, B. S. (2015). Parents' challenges and physicians' tasks in disclosing cancer to children. A qualitative interview study and reflections on professional duties in pediatric oncology. *Pediatric Blood & Cancer, 62*(12), 2177–2182.

59 Malek, op. cit. note 26, p. 180.

60 Last & van Veldhuizen, op. cit. note 58; Masera et al., op. cit. note 1; Coyne et al., op. cit. note 7.

61 Lewis, C. C., Pantell, R. H., & Sharp, L. (1991). Increasing patient knowledge, satisfaction, and involvement: Randomized trial of a communication intervention. *Pediatrics, 88*(2), 351–358; British Medical Association, op. cit. note 1.

62 Last & van Veldhuizen, op. cit. note 58.

63 Ibid.

64 Young, B., Dixon‐Woods, M., Windridge, K. C., & Heney, D. (2003). Managing communication with young people who have a potentially life threatening chronic illness: Qualitative study of patients and parents. *BMJ, 326*(7384), 305–308.

65 Bates, A. T., & Kearney, J. A. (2015). Understanding death with limited experience in life: Dying children's and adolescents' understanding of their own terminal illness and death. *Current Opinion in Supportive and Palliative Care, 9*(1), 40–45, p. 42.

66 The distinction between overall best interests and medical best interests seems to be most common in relation to judicial decisions in the United Kingdom. We take the position that there is not a useful distinction between medical best interests and overall interests, as doctors and other clinicians are ethically obliged to take into account and promote all the interests of a child, not just those that might be encompassed under “medical interests.” This is a common position in the literature. See, for example: Cave, E., Brierley, J., & Archard, D. (2020). Making decisions for children—Accommodating parental choice in best interests determinations: Barts Health NHS Trust v Raqeeb [2019] EWHC 2530 (Fam); Raqeeb and Barts Health NHS Trust [2019] EWHC 2531 (Admin). *Medical Law Review, 28*(1), 183–196; Foster, C. (2019). The rebirth of medical paternalism: An NHS Trust v Y. *Journal of Medical Ethics, 45*(1), 3–7; and Baines, P. (2010). Death and best interests: A response to the legal challenge. *Clinical Ethics, 5*(4), 195–200.

67 We thank the anonymous reviewer for this term.

